# Recursive N-Way Partial Least Squares for Brain-Computer Interface

**DOI:** 10.1371/journal.pone.0069962

**Published:** 2013-07-26

**Authors:** Andrey Eliseyev, Tetiana Aksenova

**Affiliations:** CLINATEC, CEA, Grenoble, France; University of Adelaide, Australia

## Abstract

In the article tensor-input/tensor-output blockwise Recursive N-way Partial Least Squares (RNPLS) regression is considered. It combines the multi-way tensors decomposition with a consecutive calculation scheme and allows blockwise treatment of tensor data arrays with huge dimensions, as well as the adaptive modeling of time-dependent processes with tensor variables. In the article the numerical study of the algorithm is undertaken. The RNPLS algorithm demonstrates fast and stable convergence of regression coefficients. Applied to Brain Computer Interface system calibration, the algorithm provides an efficient adjustment of the decoding model. Combining the online adaptation with easy interpretation of results, the method can be effectively applied in a variety of multi-modal neural activity flow modeling tasks.

## Introduction

The Brain Computer Interface (BCI) is a system which translates recordings of the brain’s neural activity into commands for external devices. For neuronal signal decoding, a control model is adjusted to an individual brain during the BCI system learning. This is called calibration. The model allows the control of an external effector at the stage of the online execution. Multi-way analysis was recently reported [1]–[8] to be an efficient way to calibrate BCI systems by providing simultaneous signal processing in several domains (temporal, frequency and spatial). Multi-way analysis represents a natural approach for modalities fusion [9]. It is based on the tensor data representation. Several tensor-based approaches have been invented (PARAFAC [10], Tucker [11], Non-negative Tensor Factorization [12], Sparse Nonnegative Tucker Decomposition [13], General Tensor Discriminant Analysis [14], Regularized Tensor Discriminant Analysis [7], etc.). Among them, a Multi-way or N-way Partial Least Squares (NPLS) regression was invented [15], [16] as a generalization of ordinary Partial Least Squares (PLS) [17] for the case of tensor variables.

One of the major problems in BCI studies is the variability of the neuronal signals, in particular, due to brain plasticity. The changes in the neuronal activity require recalibration of the BCI systems. The full system recalibration is a time and labor-consuming procedure. Adaptive calibration aims to provide a fast adjustment of the BCI system in response to mild changes of the signal. For adaptive modeling the online (consecutive) algorithms are applied. In addition, consecutive algorithms are efficient tools to treat the signal for a long period of observation. Let us note that the task of calibration of the BCI system analyzing the signal in several domains (temporal, frequency and spatial) is characterized by significant duration of observation as well as by huge feature space dimensions. For the high dimensional observations (more variables than observations) Partial Least Squares modeling is particularly suited [17]. A blockwise Recursive Partial Least Squares [18] allows online identification of Partial Least Squares regression. N-way PLS (NPLS) [15] provides a generalization of ordinary PLS to the case of tensor variables. Similarly to the generic algorithm, NPLS combines regression analysis with the projection of data into the low dimensional space of latent variables using tensor factorization. The blockwise Recursive PLS adapted to multi-way structured inputs (tensor-input scalar-output) was invented by the authors recently [19]. This article presents blockwise Recursive NPLS (RNPLS) extended to the most general case of tensor-input/tensor-output, its numerical study, testing and comparison. The algorithm is addressed to a data set of huge dimensions and conducts a sequential blockwise tensor-data processing. Unlike the multi-pass blockwise Iterative NPLS [8], which repeatedly runs through the entire data set, the new RNPLS algorithm performs a consecutive calculation and can be applied online. Moreover, in the case of non-stationary tensor-valued processes, RNPLS allows adaptive learning by introducing the forgetting factor.

Extending the results published in [19], the present article generalizes RPLS to the case of tensor-input and tensor-output variables. Moreover, it studies the convergence of regression coefficients as well as their variance and expectation and compares them with “true” ones. Finally, the computational efficiency and the generalization ability are compared for a set of PLS family methods (RNPLS, generic NPLS, multipass blockwise Iterative N-way Partial Least Squares (INPLS), Unfolded PLS (UPLS)). For this purpose, a set of computational experiments were carried out.

Finally, the generalization abilities of the algorithms are examined using recordings from real-life BCI preclinical experiments. In particular, RNPLS is applied to solve the tensor-input and scalar-output problem of binary events prediction in long-term experiments in freely moving animals.

Generalization of the algorithm allows us to consider another problem of continuous hand movement trajectory reconstruction from Electrocorticogramm (ECoG) recordings: simultaneous prediction of 3D coordinates of the hand at three points (wrist, elbow, shoulder).

## Methods

The RNPLS algorithm unites the recursive calculation scheme of Recursive PLS regression with the multimodal data representation of NPLS.

### Generic PLS

Ordinary PLS [17] is an iterative procedure to model a linear relationship between vector input and output variables on the basis of matrices of observations, 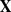 and 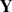: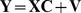, where 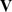 and 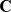 are noise and coefficient matrices respectively. PLS is a statistical method particularly suited to the high dimensional observation vectors. In order to build the model, the observations are projected into the low dimensional spaces of latent variables in such a way that the maximum variances of 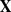 and 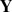 are explained simultaneously. At the first iteration, the matrices 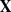 and 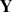 are represented as 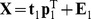, 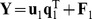, where 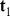 and 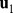
are the latent variables (score vectors), while 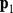 and 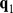 are the loading vectors. 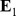 and 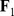 are the matrices of residuals. The score vectors are calculated to maximize the covariance between 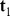 and 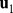. The coefficient 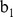 of a regression 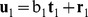 is calculated to minimize the norm of the residuals, 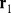. Then the procedure is applied iteratively 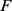 times to the residual matrices. It is demonstrated in [18] that the latent variables could be constructed as orthonormal:
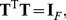
(1)where 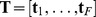, 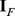 is identity matrix.

### Generic NPLS

NPLS is an algorithm of the PLS family adapted to multimodal data (tensor variables). Tensors, or multi-way arrays, are higher-order generalizations of vectors and matrices. Elements of a tensor 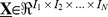 are denoted 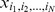. Here, 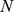 is the order of the tensor, i.e., the number of dimensions, also known as ways or modes. Vectors and matrices are tensors of order one and two respectively. The number of variables 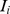 in the mode 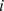 shows the dimensionality of the mode (for more details, see [20]). The tensor 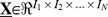 can be unfolded into a matrix along the 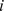 *-th* mode [20]. In this paper this operation of unfolding along the *i-th* mode is referred to as 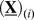. The vectorization of tensor 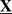 is denoted as 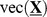 [20].

NPLS models a linear relationship between input and output variables on the basis of tensors of observations 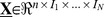 and 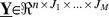. The first dimension of the array corresponds to the points of observation, while other dimensions are related to the modes of analysis (e.g., time, frequency, and space).To project data into the low dimensional feature space, NPLS uses the tensors decomposition with a set of projectors corresponding to each modality. At the first iteration, the tensors 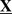 and 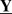 are represented as.
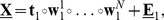


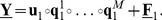
(2)


The operation “

” is the outer product [20]. The latent variables 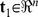 and 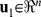 are extracted from the first mode of the tensors 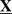 and 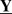, providing maximum covariance between 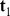 and 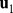. In parallel, the algorithm forms the factors, i.e. the sets of vectors 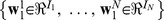, 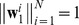, and 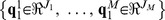 related to corresponding modes of 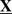 and 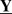. They are found in such a way that projection of the tensor 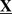 on the vectors 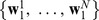 results in 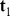 and projection of the tensor 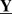 on the vectors 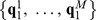 results in 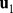. The coefficient 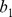 of regression 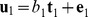 is calculated by least squares. Next, factors are calculated in the same way by decomposing the residuals 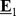 and 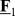. NPLS latent variables 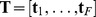 are not orthogonal. A detailed description of NPLS can be found, for example, in [16].

### Recursive PLS

The recursive PLS algorithms [18], [21], [22] were invented to take into account time-dependent changes in the data and were designed for matrices of observations. In comparison to other methods, Qin’s blockwise recursive algorithm is more efficient in updating the model with respect to the computational cost and memory [18]. To model a linear relationship between vector input and output variables 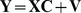 on the basis of matrices of observations, 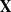 and 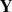 (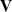, 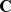 are noise and coefficient matrices respectively), Qin’s algorithm considers the decomposition of the matrices 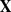 and 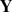 with orthonormal input latent variables 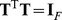:
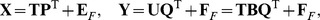
where 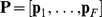, 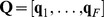 are the matrices of loading vectors, 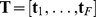 and 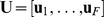 are the matrices of latent variables (score), and 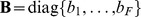 are regression coefficients for latent variables. In addition, by construction latent variables 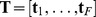 are orthogonal to residuals:
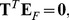
(3)

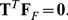
(4)


It has been shown [18] that if 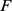 is large enough to provide 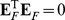, then 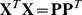 and 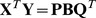. This yields that, for the new data matrices 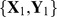, regression on the joint data sets,
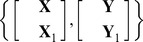
is equivalent to the regression calculated using the loading matrices (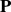 and 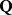) and the matrix of coefficients 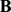 from previous observations:
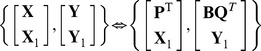



Here, the equivalence of regressions 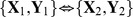 means an equality of the least square estimates of regression coefficients: 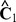 = 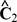, 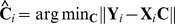 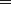 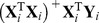, 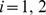.

Thus the old data sets 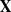 and 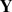 are captured by the loading matrices and regression coefficients, while the new data is added directly to them. As a result, the algorithm always keeps the size of the processing matrices.

Blockwise recursive PLS was applied for the adaptive modeling and monitoring of time-varying and non-stationary processes with slow and abrupt changes [18], [23]–[25] by introducing the sliding window and/or forgetting factor.
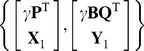
(5)The forgetting factor 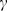 is either fixed over the entire range of 0 to 1, or estimated dynamically by assessing changes in the current data [24].

### Recursive NPLS

To apply the recursive approach to the NPLS algorithm, orthonormality of latent variables 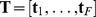 as well as their orthogonality to residuals should be provided (see Equation 1, Equation 3 and Equation 4). Let us note that the NPLS latent variables do not satisfy these conditions. The modification of NPLS to meet the requirements of Equation 1, Equation 3, and Equation 4 integrated to a recursive algorithm is described below.

Similar to recursive PLS, RNPLS is a sequential blockwise algorithm. Data blocks are received and then processed. The first block of data, namely tensors 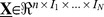 and 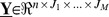, is factorized with NPLS. The procedure results in the sets of projectors 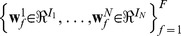, 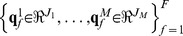, as well as the matrix of latent variables 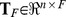 and regression coefficients 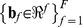 (Algorithm S1, steps 4–17). It provides the approximations 
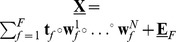
 and 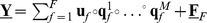.

For the next steps, we use the matrix representation. It does not affect the proposed approach, but simplifies the notation. To convert to the matrix form, the tensors 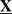 and 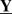 are unfolded along the first mode into the matrices 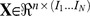 and 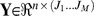: 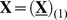, 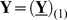. At the same time, let us denote the vectorization of the tensors 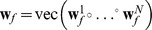, 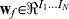, 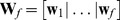 and 
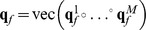
, 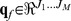 (Algorithm S1, steps 18–21). In the matrix notation 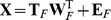, 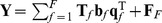.

In order to use Qin’s approach, the orthogonality of latent variables 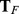 is required (see Equation 1). Since the NPLS latent variables 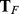 are not orthogonal, let us orthonormalize them 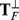: 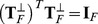. It is constructed in a way to provide additional orthogonalization of 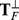 to the residuals matrix 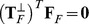. 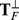 can be obtained, for example, from a Gauss orthogonalization procedure [26]. For the new orthonormal latent variables 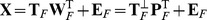, where 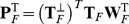 (see Algorithm S1, steps 22–25). After the orthonormalization, Equation 1 and Equation 4 are satisfied, i.e., 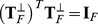 and 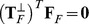.

To provide the orthogonality of the matrix 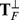 and the residual matrix 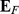 (Equation 3), let us subtract from the residual matrix its projection on all latent variables 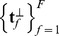: 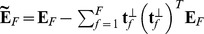. Thus, 
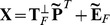
, with a new matrix of loadings 
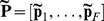
, 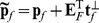. The relation 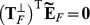 now holds. Let us define 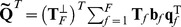 (see Algorithm S1, steps 26–30).

Since Equation 1, Equation 3 and Equation 4 are satisfied, then 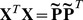, 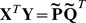. Hence, the least squares coefficients are equivalent for the data sets [18]:
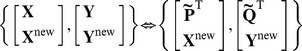



In tensor notation (see Algorithm S1, steps 31–33):
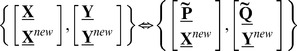



Here, a tensor 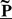 is obtained from the matrix 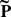, with 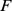 as the dimensionality of the first mode. The dimensions of the other modes are equal to the dimensions of the corresponding modes of 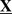. Similarly 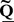 is obtained from the matrix 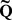 according to the dimensions of the corresponding modes of 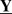.

**Figure 1:**
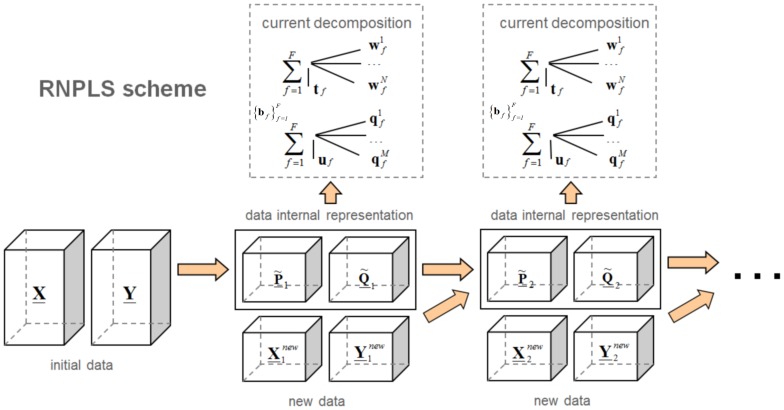
The RNPLS scheme. Information from the tensors of observation 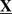 and 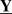 of order 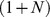 and 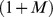, respectively, is captured by their loading tensors 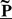 and 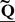. On every iteration, the algorithm generates the current sets of the coefficient vectors 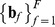, as well as the projection vectors 
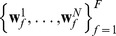
 and 
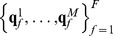
 used to estimate the dependent variable 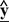 on the prediction stage.

Thus, the RNPLS algorithm inherits the tensor representation of the data and allows consecutive learning, which is a property of recursive PLS. Besides tensors 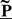 and 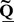 for recursive calculations, the sets of projectors 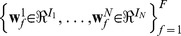, 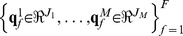 and coefficients 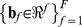 are identified. They are used at the prediction stage to estimate the output 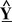 in the same way as it is done in the traditional NPLS [15]. A graphical representation of the RNPLS algorithm is shown in Figure 1.

All schemes of adaptive modeling developed for the matrix RPLS can be directly applied to adaptive learning of RNPLS. For instance, similarly to Equation (5) we can introduce the forgetting factor 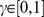:
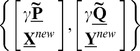



### Simulation Study

Both the generalization ability (prediction error) and the convergence of model coefficients were studied in simulation experiments. The resulted prediction errors of the proposed approach were compared with ones generated by other state of the art methods.

The computational experiments were performed on simulated datasets for matrix input and scalar output, the simplest case for simulation and comparison of tensor based algorithms. Keeping the essential properties, it simplifies the visualization and interpretation of results.

A set of PLS family offline algorithms for tensor data analysis, namely, generic NPLS, Iterative NPLS, and unfolded PLS, were compared with new sequential RNPLS. Let us note that all these algorithms generate the set of projectors and the set of coefficients of linear regression in low dimensional spaces of latent variables to predict the output variable from the input one. This model can be rewritten in the original variables. In the particular case of scalar 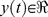 and matrix 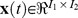,
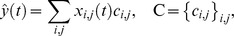



The matrix of coefficients 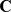 is estimated by each algorithm. To analyze the convergence properties of sequential blockwise RNPLS, the training data were split into 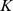 disjoint subsets. The RNPLS algorithm was consequently applied to these subsets, i.e., each following subset was used to adjust the solution from the previous step. 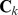 is the approximation of the regression coefficient matrix estimated at step 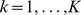.

The variance and expectation of the coefficients evaluated by the different methods were examined in comparison with “true” coefficients. Prediction errors of algorithms mentioned above were compared on simulated datasets. Furthermore, the adaptive properties of the RNPLS-based adaptive algorithm to abrupt changes in the observed process were evaluated.

**Figure 2:**
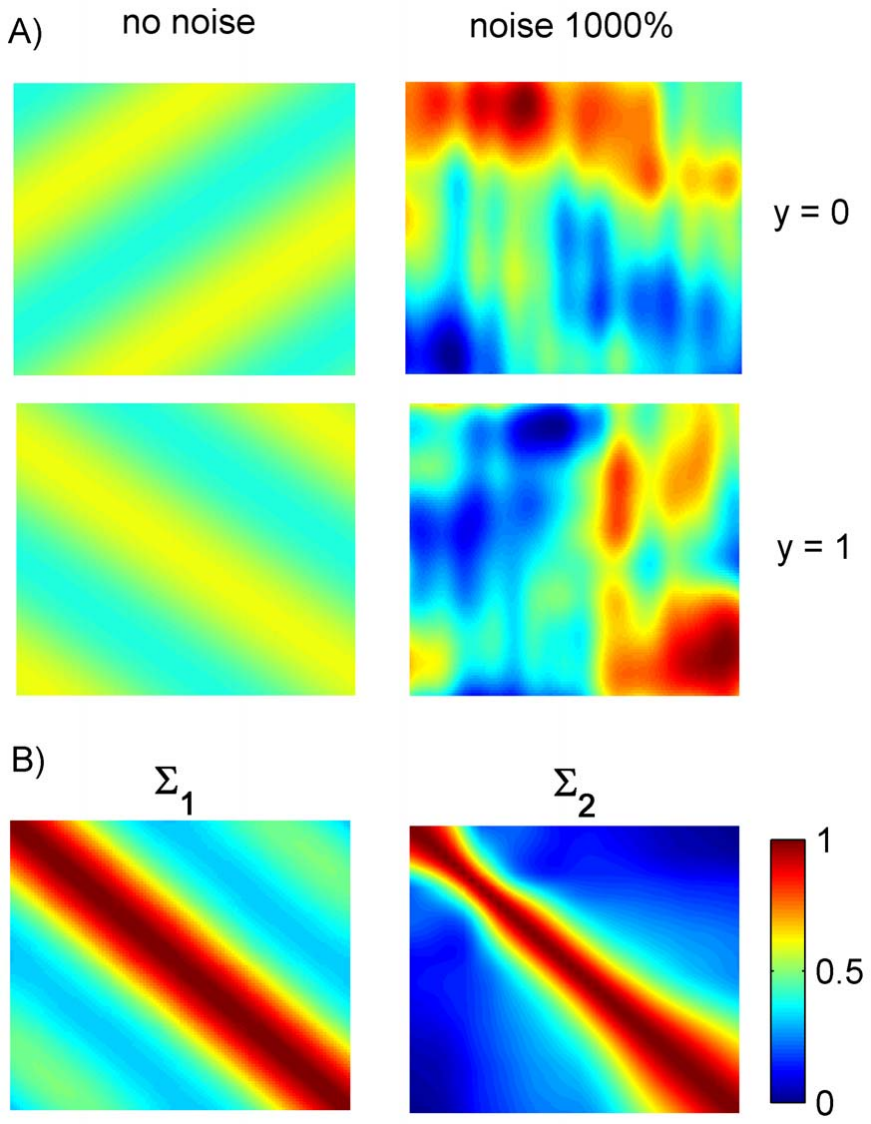
Artificial data. A) Points 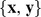 without noise and with 1000% of noise (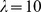) from the simulated data set. B) Covariance tensor 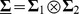 to generate samples of the simulated data set is defined as a Kronecker product of covariance matrices 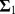 and 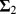. Matrices 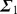 and 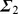 were estimated from experimental data (Section *Binary BCI*) as correlation matrix for the temporal and frequency modalities in the most informative time interval [0, −1.5] s and frequency band [50, 300] Hz.

The tests were performed for the binary output variable which corresponds to the binary BCI experiment described below (see details in [8]). Despite its simplicity, this model makes it possible to explore the fundamental properties of the method. An artificial data set 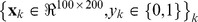
was generated following [19]. Namely, binary outputs 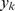 were randomly generated (0 or 1) with equal probabilities. Matrices of input 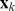 (Figure 2A) were calculated according to 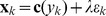,
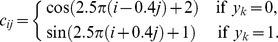



The random noise 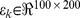 was drawn from a multivariate normal distribution 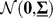. To take into account the covariance structure of the real data, the covariance tensor 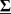 was defined as a Kronecker product of two matrices 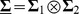 where the matrices 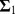 and 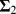 were estimated as covariance matrices of the temporal and frequency modalities of the real brain ECoG activity recordings (binary BCI is described below in the Section *Binary BCI*). Correlation matrices are shown in Figure 2B. Thus the artificial data inherit some of the statistical properties of the real recordings. The same noise distribution was applied for both classes in the simulated data. The noise 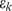 was added to the templates with coefficients 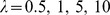. It has the same amplitude as the signal 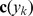 if 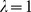.

In the particular case of this computational experiment, the matrix of regression coefficient 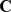 belongs to 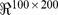.

### Convergence of RNPLS Regression Coefficients

Generally speaking, the convergence of the output variables 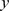 does not require the convergence of the regression coefficients 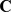 so far as the convergence of the regression coefficients is a stronger requirement.

To analyze the convergence of regression coefficients, we examined the distance between 2 matrices 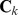 and 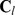,
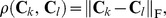
where 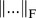 is the Frobenius norm.

In addition, to evaluate the RNPLS regression coefficients, they were compared with the “true” ones. The “true” coefficients are estimated using the simulated data without noise (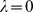). In the particular case, the difference of the centers of the classes represents the matrix of regression coefficients (up to a scaling factor and a constant term). They could be evaluated, for instance, by the ordinary PLS.

**Figure 3:**
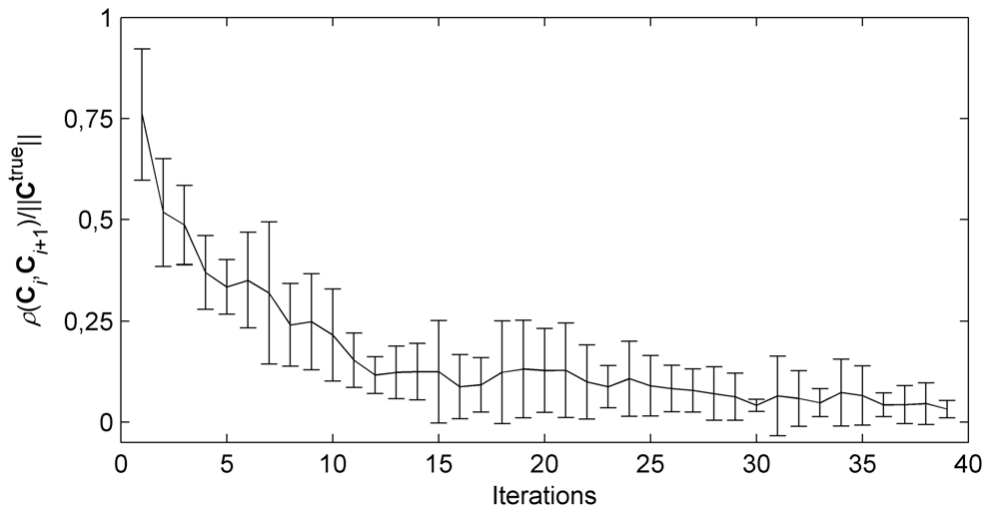
Convergence of the regression coefficients matrices. Convergence of the coefficients matrices versus iterations 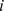 (mean and standard deviation) of the RNPLS algorithm in the series of experiments (10 realizations, simulated dataset, level of noise 1000%). The distance 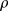 between two successive coefficient matrices decreases on ∼70% (in ∼3.5 times) after the first 12–15 iterations.

**Figure 4:**
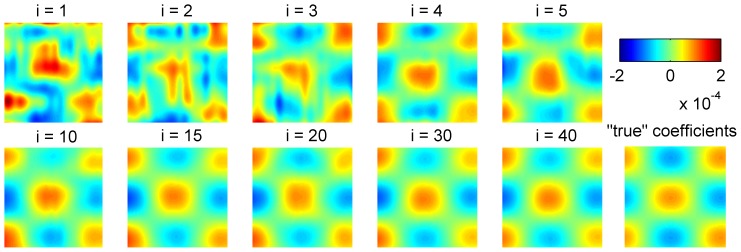
Examples of the regression coefficients. The regression coefficients 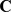 depending on the iteration number 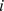 of the RNPLS algorithm in one of the computational experiments for the simulated dataset, level of noise 1000%.

To apply blockwise RNPLS, a simulated data set (800 points) was split into 40 disjoint subsets (blocks), each one containing 20 points. The level of noise was taken to be 1000% of the signal amplitude (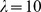). The convergence of the regression coefficient matrices for the series of computational experiments (10 realizations) is represented in Figure 3 and Figure 4. The changes in 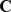 become insignificant after approximately 10–15 iterations. The number of factors, 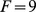, was chosen in such a way as to minimize the average root mean squared error (RMSE) on the test dataset over 10 realizations of the training dataset.

### Adaptivity

The second computational experiment was performed to verify the adaptive properties of the RNPLS. An abrupt change was introduced to the simulated data set (2000 points). Namely, the indexes of the classes were reversed from the point number 1000 for the rest of the data set. The level of the noise was chosen equal to 1000% (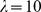). 10 realizations of the noise were generated.

**Figure 5:**
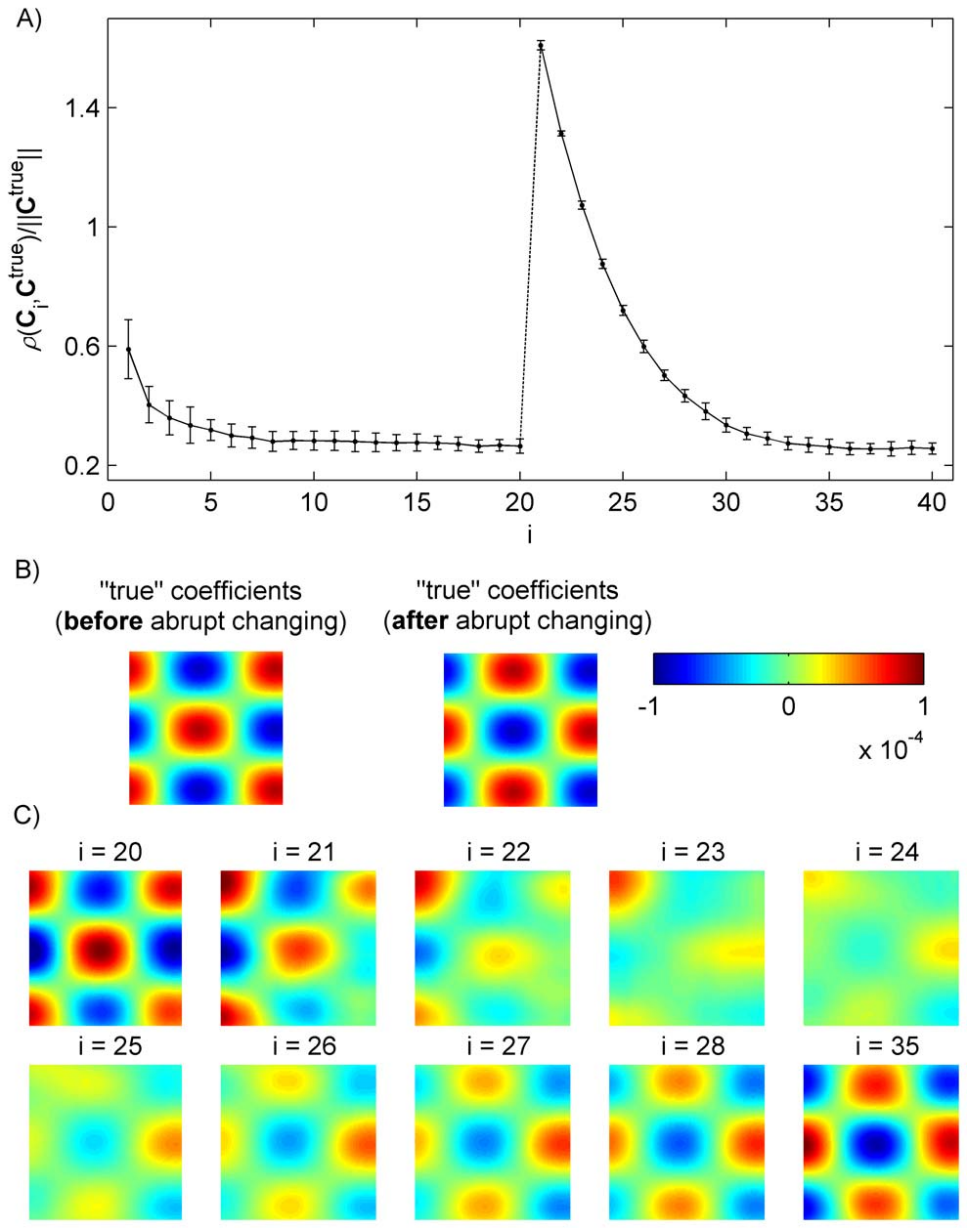
Adjustment of the RNPLS regression coefficients to abrupt changes in observations. A) The distance 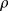 (mean and standard deviation) between the RNPLS and “true” coefficients versus the iterations of the RNPLS algorithm in the series of experiments (10 realizations of the simulated dataset, level of noise 1000%). The solution of the RNPLS algorithm is adjusted in 15 iterations to the abrupt changes in observation (at *21^st^* iteration) with the forgetting factor 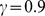. B) “True” coefficients before after abrupt changes at *21^st^* iteration. C) Example of adjusting the regression coefficients over iterations 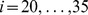.

To apply adaptive blockwise RNPLS, the data was split sequentially into blocks containing 50 points. The same number of factors (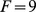) was applied. For the first 20 blocks (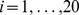), the algorithm successfully converged to “true” regression coefficients. For the next subsets (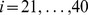) the classes and, therefore, the “true” regression coefficients were reversed. Figure 5 demonstrates that the RNPLS algorithm adjusted its solution to the new situation in 15 iterations (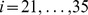) (forgetting factor 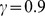).

### Comparison of Algorithms

To study whether the recursive scheme of the RNPLS algorithm decreases the accuracy of modeling, the proposed method was compared with generic offline NPLS, iterative NPLS and unfolded PLS on the simulated data. The percentage of prediction errors (RMSE) was estimated depending on the level of noise and the number of factors. The variance of the coefficients was studied for the set of algorithms. Finally these coefficients were compared with the “true” ones.

The entire simulated dataset (1600 points) was equally split into the training and the test datasets. The NPLS, iterative NPLS, and unfolded PLS algorithms processed the whole training set. For the recursive calculation, the training dataset was split into 20 disjoint windows, each one containing 40 points. For all conditions (different noise level and the number of factors), the experiment was repeated 10 times with new realizations of noise.

The percentage of prediction errors was estimated using 10 realizations of the test dataset depending on the level of noise and number of factors for all the algorithms. RNPLS demonstrated a significantly smaller number of factors which are necessary for efficient prediction (Table 1). Let us remember that the RNPLS showed better robustness, as the variation of the prediction errors was essentially smaller for the recursive algorithm for a small number of factors [19].

**Table 1:** Comparison of the optimal number of factors and RMSE for RNPLS, NPLS, INPLS, and UPLS.

	RNPLS		NPLS				INPLS				UPLS			
Noise, %	Number of factors	RMSE, %	Number of factors	RMSE, %	*t*	*t*_0.01_	Number of factors	RMSE, %	*t*	*t*_0.01_	Number of factors	RMSE, %	*t*	*t*_0.01_
50	8	0.16±0.01	15	0.15±0.01	2.23	2.88	14	0.14±0.01	4.47	2.88	14	0.15±0.01	2.23	2.88
100	10	0.32±0.03	17	0.29±0.01	3.00	3.12	15	0.30±0.02	1.75	2.93	16	0.30±0.02	1.75	2.93
500	8	1.6±0.2	15	1.5±0.1	1.41	3.00	9	1.5±0.1	1.41	3.00	11	1.5±0.1	1.41	3.00
1000	9	3.1±0.2	17	2.8±0.1	4.24	3.00	17	3.0±0.3	0.88	2.93	13	2.9±0.2	2.24	2.88

The difference of RMSE for RNPLS and the other methods is not statistically significant (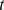
<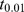) in the majority of the cases (10 from 12) according to the two-tailed Welch’s 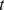-test for 1% confidence level 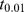.

**Figure 6:**
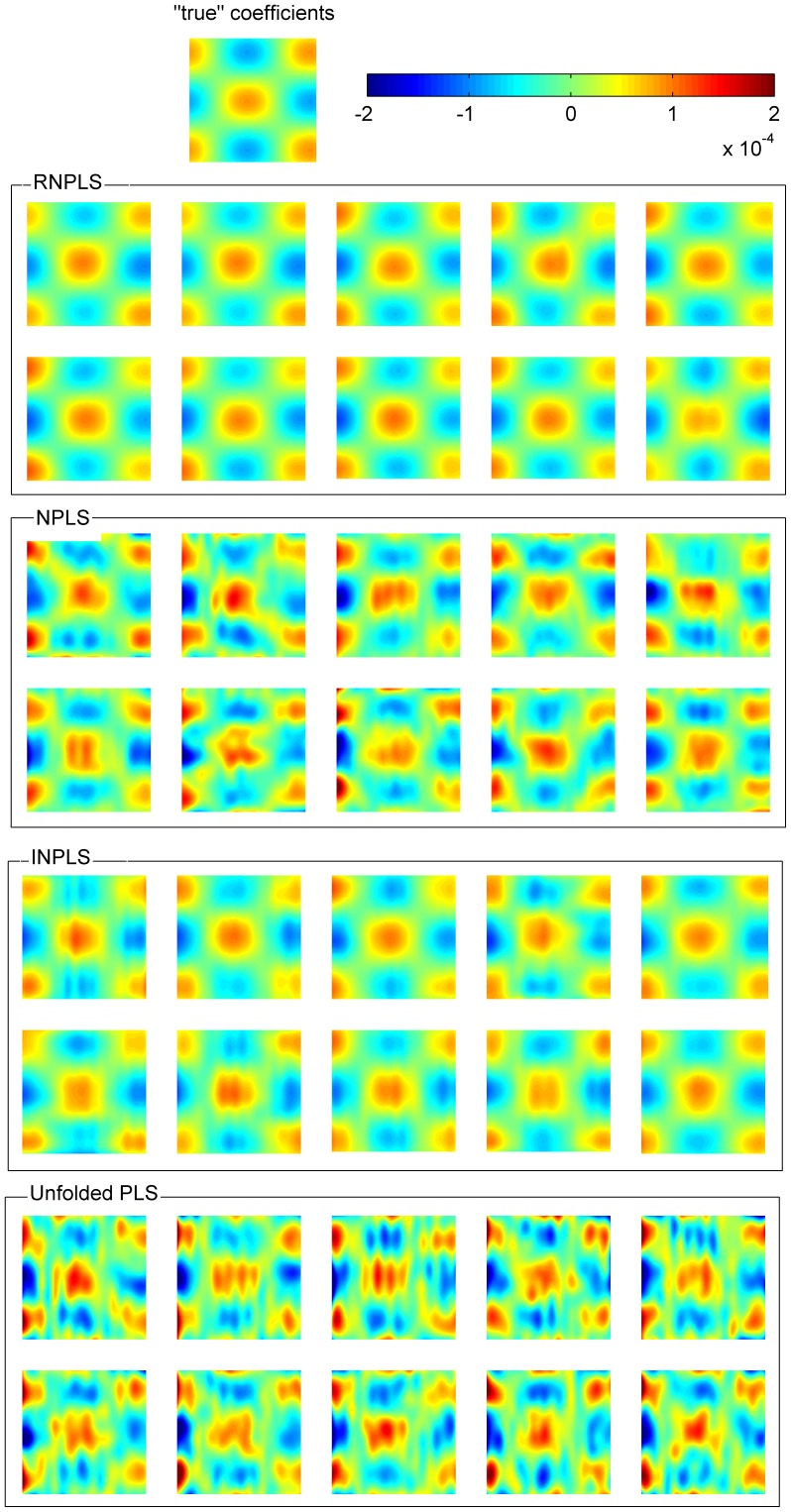
Examples of regression coefficients for different methods. RNPLS, NPLS, INPLS and UPLS regression coefficients for 10 realizations of the simulated training dataset (level of noise 1000%).

To compare the variance of the estimated coefficients, RNPLS, NPLS, INPLS, and UPLS algorithms were applied to 10 realizations of the training dataset with the level of noise 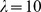 (1000%). The factor numbers were chosen equal to 9, 17, 17, and 13, for RNPLS, NPLS, INPLS, and UPLS, respectively, in order to minimize the average RMSE in the 10-fold cross-validation procedure. For this procedure, the training set is randomly partitioned into 10 equal size subsets. 9 subsets are used for training, whereas one is used for the test, i.e., RMSE calculation. The process is then repeated 10 times and the 10 results are averaged to produce a single RMSE estimation. The whole procedure is repeated for the different number of factors to find the one which minimizes RMSE. For each calibration approach, for the optimal number of factors, the images of the obtained regression coefficients 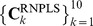, 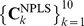, 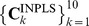 and 
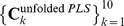
 are represented in Figure 6. The variability of the unfolded PLS results (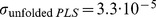) is almost 5 times greater than the variability of the RNPLS results (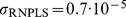), whereas the variabilities of INPLS and NPLS are 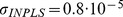 and 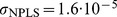, respectively.

**Figure 7:**
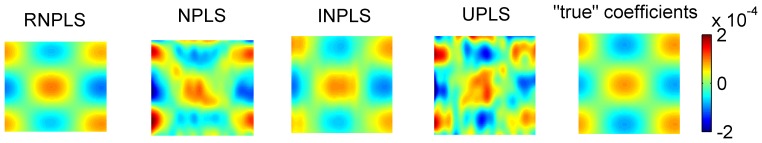
The RNPLS, NPLS, INPLS, UPLS and “true” regression coefficients. Comparison of the regression coefficients averaged over 10 realizations of noise in the simulated dataset (level of noise 1000%). RNPLS, NPLS, INPLS, UPLS and “true” coefficients.

**Figure 8:**
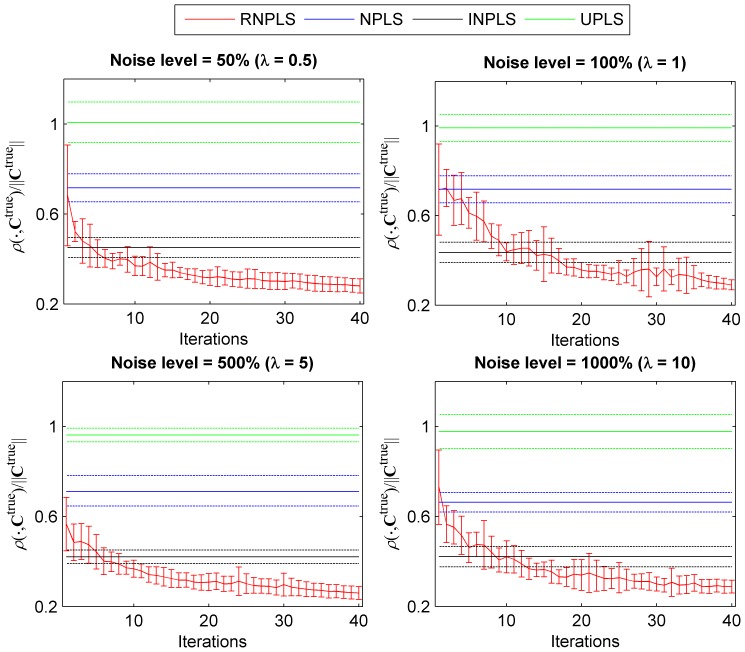
Comparison of regression coefficients for different levels of noise. The mean values and the standard deviations (10 realizations of random noise in the simulated dataset) of the distance between the “true” regression coefficients and RNPLS regression coefficients 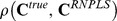 over the RNPLS recursive iterations (red lines); between the “true” coefficients and regression coefficients generated by the NPLS applied to whole training datasets 
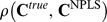
 (blue lines); between the “true” coefficients and regression coefficients generated by INPLS (black lines) and by UPLS (green lines).

“True” coefficients (PLS without noise), the regression coefficients for RNPLS, NPLS, INPLS, and UPLS methods (averaged over 10 realizations, 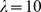) are shown in Figure 7. It can be seen that the result generated by RNPLS is closer to the “true” coefficients than the NPLS, INPLS, and UPLS solutions. To estimate the difference numerically, the mean values and standard deviations of 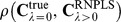 for different noise levels 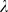 were calculated over the algorithm iterations (Figure 8). In parallel, the means and the standard deviations of 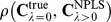, 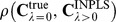 and 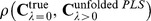 were computed for every level of noise. All statistics were calculated for 10 realizations. As can be seen, the mean value of the distances between the RNPLS and “true” regression coefficients after 15 iterations (the first 20–100 points of the training set) is less than those between the NPLS, or UPLS and “true” regression coefficients computed for the whole training datasets (800 points), whereas RNPLS needs up to 14 iterations (280 points) to outperform the INPLS results. In addition, the standard deviations of the RNPLS results are generally less than those obtained by NPLS, INPLS, or UPLS.

The advantages of RNPLS can be explained by overfitting suppression.

### Application

The particular goal of our study is to develop an efficient algorithm for BCI system calibration. The RNPLS algorithm was tested on real data sets recorded during BCI experiments.

### Binary BCI

Both RNPLS and generic NPLS algorithms were applied in parallel to calibrate the binary BCI system. The data set was collected during the binary self-paced BCI experiments on a freely moving rat.

The experiments were carried out by the neurophysiological team at CEA, LETI, CLINATEC. Behavioral experiments were based on a reward-oriented task. A rat freely moving in a cage had the opportunity to push a pedal to activate a food dispenser. The BCI task consisted in detection (prediction) of the pushing event by analyzing the recording of the rat’s neuronal activity (see details in [8]). During BCI experiments the Electrocorticogram (ECoG) (the signal from 14 electrodes located on the surface of the cortex) and the signal of the pedal were recorded simultaneously, downsampled to 1.3 kHz and band-pass filtered between 0.5 Hz and 500 Hz. The Common Average Reference (CAR) filter was applied to eliminate a “common source” [27].

To form a feature tensor, each ECoG epoch was mapped to temporal-frequency-spatial space by the continuous wavelet transform (CWT). For each epoch 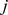 (determined by its final moment 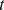), electrode 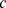, frequency 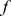 and time shift 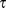, elements 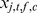 of the tensor 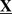 were calculated as norm of CWT coefficients. The frequency band 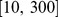 Hz with step 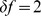 Hz and sliding windows 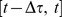, 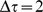 s with step 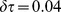 s, were considered for all electrodes 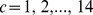. The resulting dimension of a point was 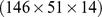. The binary dependent variable was set to one, 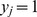, if the pedal was pressed at the moment 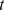, and 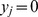 otherwise.

For the model identification, the tensor of observations was formed from a 10 minute long recording. The tensor included 400 points corresponding to event-related epochs (50 pressing events repeated 8 times) and randomly selected 1000 non-event epochs. The tensor of observation was split into the training and test tensors. Namely, 1000 randomly selected points (700 “non-events” and 300 “events”) formed the training dataset, while 400 points (300+100) were used for the test. The proportion of the classes is chosen to avoid a class imbalance problem (e.g. [28]). Generally, binary self-paced experiments [29] lead to imbalance classes: the “minority” class has a very small number of examples (class of events), while the “majority” class includes a large number of cases (class non-event). Most of the machine learning approaches are strongly biased toward the majority class (e.g. [28] while the correct classification of samples from the minority class is the most important. At the same time, the non-event class should be well presented for efficient algorithm learning. To avoid the problem, the classes were formed in a less biased way, following [8].

**Figure 9:**
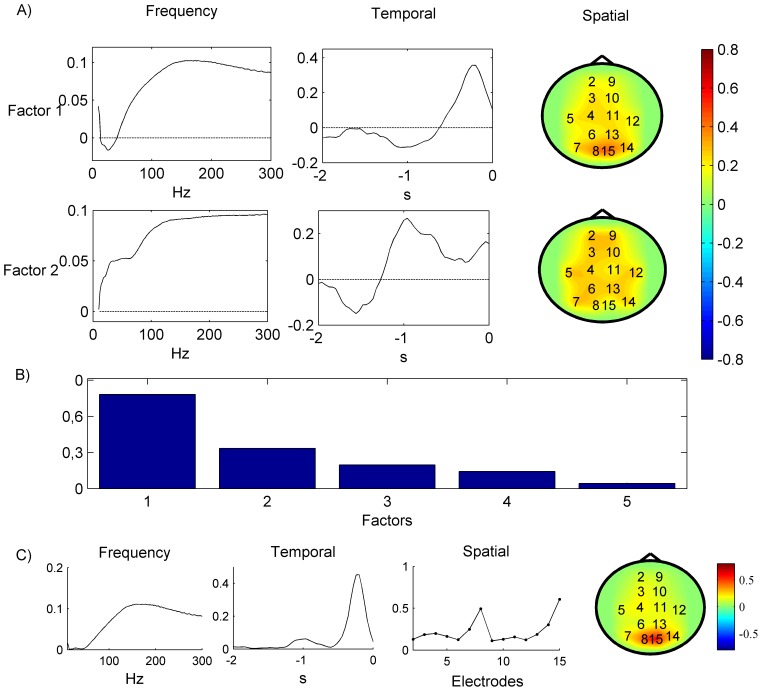
RNPLS (10) calibration. A) The first and the second factors (which are the most contributive out of 5): frequency, temporal and spatial projections. The values of elements of the spatial projector are shown in colors according to the color bar and positions of the electrodes are indicated by numbers. B) Factor weights in the final decomposition which are the coefficients 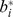 of the normalized model in the space of latent variables. C) Impact on the predictive model of different modalities’ components according to MI analysis; the spatial modality is represented by the graph and the corresponding color map. Recordings from binary self-paced BCI experiments in freely moving rat.

**Figure 10:**
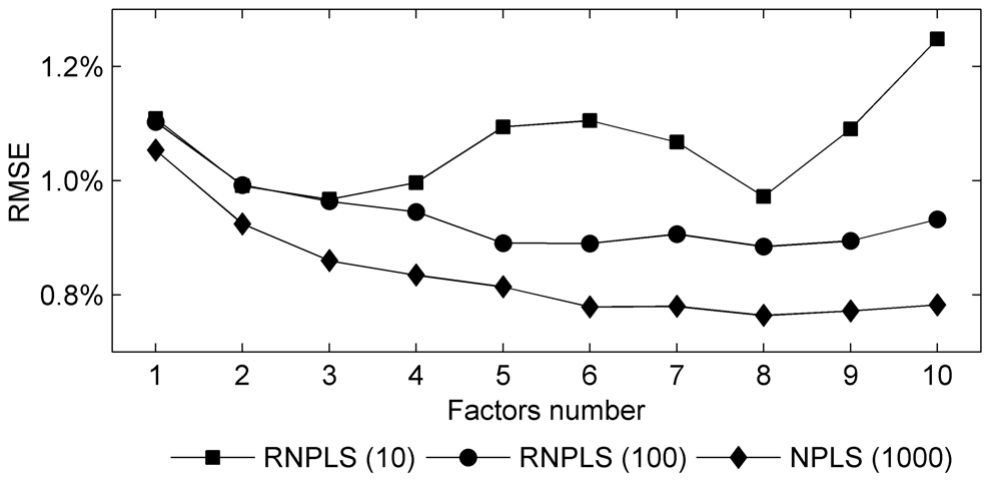
Comparison of prediction error RNPLS vs. NPLS. RMSE as a function of the number of factors for the test dataset. RNPLS (10) – the training set is split into 10-point disjoint subsets, RNPLS (100) – the training dataset is split into 100-point disjoint subsets, NPLS (1000) – generic NPLS using the whole training dataset. Recordings from binary self-paced BCI experiments in freely moving rat.

To test RNPLS, the training dataset was split into disjoint subsets with 10 and 100 points, marked as RNPLS (10) and RNPLS (100), respectively. Next, the projectors and the predictive models were identified. Figure 9A represents the first 2 factors calculated by RNPLS (10). The total number of factors was estimated to be 5 by the 10-fold cross-validation procedure. Each factor consists of 3 projectors, corresponding to frequency, temporal, and spatial modalities, respectively. Absolute values of the projector’s elements define the influence of these elements on the identified model. The weights of factors in the decision rule are different. The relative weights of factors in the final decomposition (coefficients 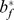, of the normalized model 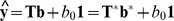, 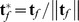, 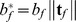, 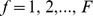) are demonstrated in Figure 9B. Figure 9C shows the summarized influence of the elements for different modalities on the predictive model according to Modality Influence (MI) analysis [30]. For instance, in the spatial modality the electrodes number 15 and 8 have the highest impact on the decision rule. In the frequency modality the main contribution is given by the frequencies band [150, 250] Hz. The most significant interval in the temporal domain is [−0.4, −0.1] s. Figure 10 shows predicted root mean squared errors (RMSE) as a function of the number of factors. With respect to NPLS, the RNPLS algorithm demonstrates minimal deterioration in the prediction quality for all numbers of factors: for RNPLS (100) it is about 0.1%, while for RNPLS (10) it is about 0.2%. Thus, the experiments demonstrated that the size of the analyzed data block could be significantly reduced without essential deterioration of the prediction quality.

### Continuous BCI

The RNPLS method was also applied for decoding of the continuous three-dimensional hand trajectories from epidural ECoG signals of a Japanese macaque. The data is publicly available (http://neurotycho.org/data/20100802s1epidural-ecogfood-trackingbkentaroshimoda). The ECoG signals were recorded (Blackrock Microsystems, Salt Lake City, UT, USA) with a sampling rate of 1 kHz from the 64 electrodes implanted in the epidural space of the left hemisphere of the monkey, which was trained to retrieve foods using the right hand. The hand motion was recorded by an optical motion capture system (Vicon Motion Systems, Oxford, UK) with a sampling rate 120 Hz. The length of the experiments was 15 minutes. During the experiment, the monkey was seated in the chair with restricted head movement. The experimenter demonstrated foods at random locations at a distance of 20 cm for the monkey at random time intervals (3.8±1.0 times per minute), and the monkey grasped the foods. A precise description of the experiments can be found in [31]. In this work, the unfolded PLS was applied for the continuous three-dimensional hand trajectories reconstruction.

To test the RNPLS algorithm one recording (15 minutes, sampling rate: 1000 Hz) was chosen randomly from the data base. To train the algorithm, 5000 time epochs were randomly selected among the recorded time moments. As a result, the training dataset includes 0.5% of the entire recording. The test dataset contains 4500 random epochs (0.45% of the entire recording) of the same file out of training.

To form a feature tensor, each ECoG epoch was mapped to temporal-frequency-spatial space by CWT. The frequency band consisted of 3 sub-bands, namely, 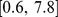 Hz with step 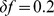 Hz, 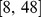 Hz with step 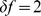 Hz, and 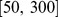 Hz with step 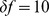 Hz. Sliding windows 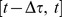, 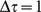 s with step 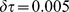 s were considered for all electrodes 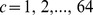. The resulting dimension of a point is 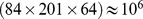 vs. 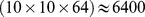 in [31] (frequency band Hz, 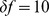 Hz, time interval 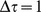 s, 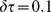 s). Since the RNPLS algorithm allows recursive data set processing, the restriction on the memory consumption is less limiting.

**Figure 11:**
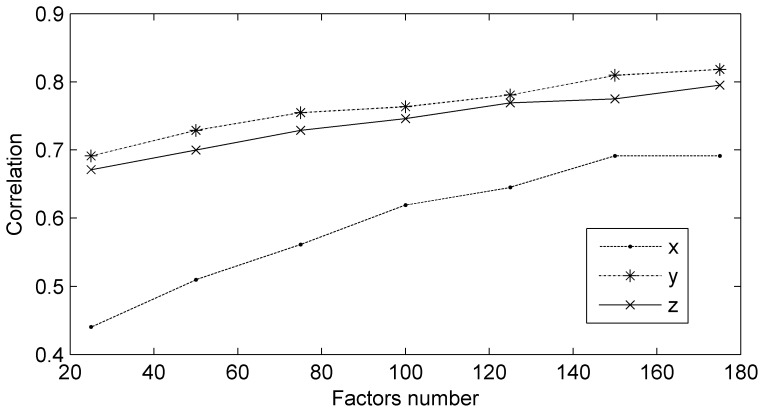
Correlation between the real and predicted coordinates. Correlation between the real and predicted coordinates as a function of the number of RNPLS factors for the test data of 3D wrist position of the monkey’s right hand.

**Figure 12:**
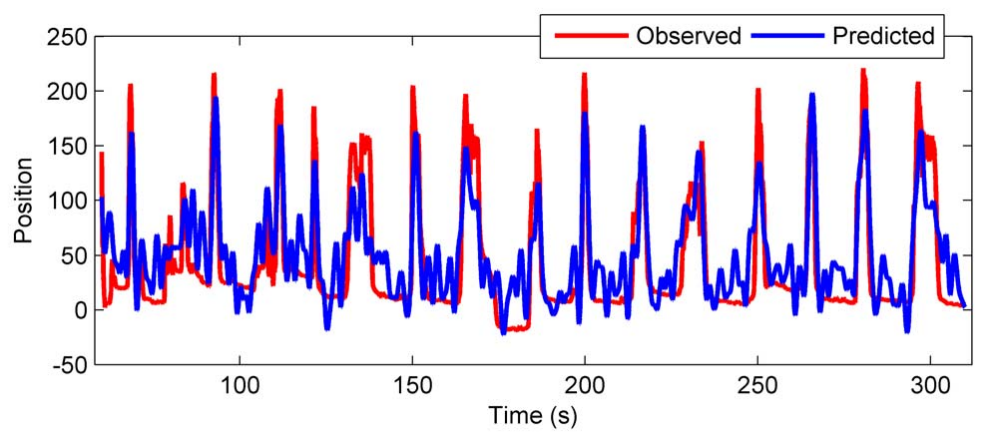
Example of the observed and predicted trajectories. Example of the observed and predicted trajectories for 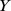-coordinate of the monkey’s right wrist. The predicted model is identified by the RNPLS approach applied for the case of tensor input and matrix output of 3D coordinates for wrist position.

Preprocessing techniques (chewing artifacts extraction, common average reference filter, etc.) were not applied. To identify the decoding model with RNPLS, the training set was split into 5 subsets of 1000 points. To validate the generalization ability, the model was applied to the test data set. Predicted wrist motion of the right hand was correlated with its real position. The resulting correlation for the test data as a function of the number of factors is shown in Figure 11. The best correlations 0.69, 0.82, and 0.79 for 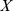-, 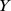-, and 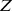-positions are achieved for 150–175 factors. An example of the real and predicted trajectories is given in Figure 12.

To test the RNPLS approach in the general case of tensor input and tensor output data, we applied the proposed method for simultaneous prediction of the monkey’s right shoulder, right elbow, and right wrist coordinates. Both the training and the test input tensors 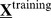 and 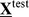 (calculated from ECoG) were not changed, whereas the output tensors 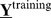 and 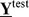 acquired one more modality (shoulder, elbow, wrist). Thus, the training dataset was 
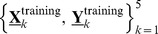
, 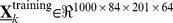, 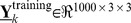 and the test dataset was 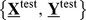, 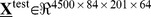, 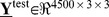. The resulting correlations between the observed and predicted coordinates are represented in Table 2. The number of factors was equal to 200. Compared with the results obtained for the wrist only, we found that predictions of the Y- and Z-coordinates are improved while the X-coordinate became slightly worse.

**Table 2:** Correlation between predicted and observed coordinates of the monkey’s right hand (test dataset, N = 4500 samples).

	*corr_x_*	*t*	*corr_y_*	*t*	*Corr_z_*	*t*
Shoulder	0.62	85.67	0.80	134.13	0.85	159.65
Elbow	0.54	72.67	0.84	153.67	0.83	148.19
Wrist	0.63	87.51	0.85	159.65	0.82	143.15

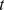
-test demonstrates the significance of the correlation (1% confidence level 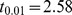).

In general, the quality of the prediction of the X-coordinates (left/right movement) for all analyzed parts of the right hand was essentially worse than prediction of Y-coordinates (backward/forward movement) and Z-coordinates (up/down movement). A possible explanation of this result is different types of muscular efforts, and therefore different levels of their representation in the ECoG recordings.

## Discussion

Neuronal signal decoding represents a challenging task. Recent promising results in the BCI area (e.g., [32]–[36]) have brought clinical applications to a realistic task. For real-life applications, the need for an easy to use BCI system is one of the crucial problems. The recursive algorithm presented in this article contributes towards solving the problem of adaptive fast and easy BCI system calibration.

The proposed RNPLS performs multimodal data analysis. While several tensor-based methods were recently efficiently applied in BCI studies, all of them are offline methods and require access to the entire data set. RNPLS unites the recursive calculation scheme with multimodal data representation and can be applied online. It uses a single-pass blockwise tensor-data processing in contrast to multipass blockwise Iterative NPLS [8], which runs over the entire recording to estimate each latent variable and corresponding set of projectors. Moreover, RNPLS allows adaptive learning by introducing the forgetting factor.

One of the important problems of sequential algorithms could be the degradation of results caused by consecutive calculation. To examine this question, the prediction accuracy of the proposed RNPLS algorithm was studied and compared with generic offline NPLS as well as two other algorithms of the PLS family (INPLS and Unfolded PLS) on artificial and real data sets. In both cases the generalization abilities of tested algorithms are comparable. We have not observed significant degradation in prediction error caused by recursive calculation.

At the same time, over the set of computational experiments recursive blockwise RNPLS provides the best approximation of the “true” model coefficients and with minimal variance. It is interesting to note that although coefficient estimates of all algorithms of the PLS family are biased, both blockwise INPLS and RNPLS provide more stable and less biased coefficients in comparison to NPLS and Unfolded PLS. While the “true” model is not necessarily the best in the sense of expectation of prediction error (e.g., [37]), the appropriate coefficient estimation is nevertheless of great importance for the interpretation of results.

Finally, the RNPLS approach demonstrates fast coefficients convergence for all tested levels of noise and is considerably noise-steady.

The recursive algorithm was compared to the generic NPLS in terms of accuracy and convergence rate on the real data of BCI experiments. In binary self-paced BCI in freely moving animals the RNPLS algorithm demonstrated minimal deterioration in the prediction quality with respect to NPLS: 0.2% using the 10 point blockwise algorithm and 0.1% using the 100 point blockwise algorithm. Treatment of smaller blocks of data does not significantly affect the generalization ability of the model. At the same time, a lower number of relevant factors was extracted by RNPLS. The 10-fold cross-validation procedure indicated 8 as the optimal number of factors for generic NPLS and only 5 factors for RNPLS. Nevertheless, taking into account the requirements for computation resources (memory), the blockwise method is favorable. The factors extracted by RNPLS algorithms are very similar to the factors reported in [8]. The same electrode has the greatest impact on the decision rule (∼16% of the extracted information for the generic approach and ∼19% for the recursive algorithm). In both cases, high frequencies [100, 300] Hz provided the main contribution to the decision (∼86% and ∼91%). In the time domain, the interval [−0.5, 0] s before the event is the most significant (∼68% and ∼78% of the influence). Let us note that less informative variables are better suppressed by the recursive algorithm. Namely, the time interval of more than 1 s before a movement is almost completely inhibited by RNPLS, which better corresponds to the physiology. Moreover, the computational efficiency of the resultant model is acceptable for real-time applications [8].

Another real-life problem in testing the recursive algorithm was the decoding of the continuous three-dimensional hand trajectory from epidural ECoG signals. The movement of the hand was represented by 3D positions of the shoulder, the elbow, and the wrist of the monkey. Thus, the motion was characterized by 9 degrees of freedom. Rigorous comparison of the proposed approach with results reported by other authors is complicated by variation of testing data. Nevertheless, the accuracy of prediction (correlations 0.63, 0.85, 0.82 for 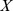-, 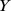-,
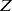
- positions of wrist) is comparable with results reported by other authors using the same or a similar data base: 0.47±0.12, 0.56±0.10, 0.68±0.06 with Unfold-PLS [31]; 0.52±0.13, 0.67±0.04, 0.74±0.04 with Higher-Order Partial Least Squares [3], [35]; 0.50±0.13, 0.67±0.04, 0.73±0.04 with NPLS [35]; 0.50±0.12, 0.67±0.04, 0.74±0.03 with Unfold-PLS [35]. The good accuracy may result from high resolution of data analyses which is achieved due to consecutive calculation. The large number of factors (150–175 vs. 36±14 in [31]) can be explained by the fact that presentation of data by tensors of order one (used in NPLS algorithms) required more factors than matrix decomposition with PLS. The continuous structure of the output signal could contribute to the significant number of factors. For instance, for the case of the binary response variables, the number of factors for the RNPLS approach did not exceed 10.

### Limitations of the Present Study and Future Work

The RNPLS algorithm applies a blockwise recursive calculation schema of Qin’s RPLS algorithm and inherits its limitations. From the theory of Qin’s recursive PLS, the number of factors 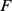 should provide a full rank of matrix of latent variables (the residuals of tensor 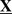 factorization should be essentially zero: 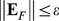, see [18]). Otherwise it could result in numerical error. RNPLS inherits this point of Qin’s algorithm. Similar to RPLS, the number of factors in Recursive NPLS should be large enough to extract the essential information. It could be chosen with cross-validation [18].

Some informative features could be weakly represented and finally lost due to insufficient size of blocks. To prevent this situation, the size of the data fragments should be adjusted depending on the number of informative factors.

In addition, the forgetting factor should be determined in accordance with the variability of the analyzed data.

The perspective of this study is preclinical (in the monkey) and clinical BCI applications. Simulation of decoding of neuronal activity demonstrates the compatibility of the predictive model with real time requirements. After efficient implementation, the algorithm will be used in the CLINATEC® BCI project which includes the realization of a fully implantable device, WIMAGINE®, to measure and transmit ECoG data wireless [38] to a terminal, and the means for a tetraplegic subject to pilot effectors, such as fragments of exoskeleton. Blockwise RNPLS allows higher resolution (frequency and temporal) of the BCI predictive model than other approaches including tensor based methods [31], [35]. In addition, it will help to take into account session-to-session BCI signal variability with fast system adjustment instead of full recalibration and to consider intra-session signal variations (degradation due to fatigue, changes caused by brain plasticity, disturbance from the outside world, etc.) The study of the algorithm’s adaptation to session-to-session, subject-to-subject variability with publicly available monkey ECoG recordings is our nearest perspective.

### Conclusion

In the current paper we consider the method of the adaptive multimodal data flow processing in the most general case of tensor-input/tensor-output data, whose properties were studied in detail for different aspects. A detailed pseudocode is provided which substantially facilitates the understanding and implementation of the proposed approach.

Contrary to other multimodal data treatment methods, such as unfolded PLS, NPLS, INPLS, etc., the RNPLS allows sequential blockwise adaptive learning. Due to significant variability of the neural data, this property is of great importance for the real-time BCI applications, where complete recalibration of the system is time-consuming and cannot be done frequently.

For the obvious demonstration of the convergence properties, they were explored on the artificial datasets. The experiment demonstrated fast and stable convergence. Tested with noisy data (the level of the noise was up to 1000%), the algorithm showed robustness and significant noise steadiness.

At the same time, the performance of the sequential algorithm is demonstrated to be comparable with the offline algorithms of the PLS family. In both simulated and real experiments, RNPLS allows one-pass online regression reconstruction without loss of generality. Note that all PLS family algorithms provide biased estimates of regression coefficients [39]. However, the recursive algorithm demonstrated better stability, lower variability and less biased coefficients, regarding the “true” ones.

RNPLS method demonstrates promising results in BCI studies. Generalization of the algorithm to the case of tensor-input/tensor-output data allows efficient continuous movement reconstruction in several points (e.g. wrist, elbow, shoulder) simultaneously.

The algorithm can be efficient in many other applications for the adaptive modeling of high dimensional tensor flows.

## Supporting Information

Algorithm S1 **RNPLS.**
(DOC)
