# Supplementary material for: Recursive N-Way Partial Least Squares for Brain-Computer Interface
Source: PLoS One. 2013 Jul 26;8(7):e69962. doi: 10.1371/journal.pone.0069962 (PMC3724854; doi:10.1371/journal.pone.0069962)
Supplement: Algorithm S1 — RNPLS. (DOC) [file pone.0069962.s001.doc]

| Algorithm S1. RNPLS |
| --- |
| *Input:* new data set $\left\{\underline{\mathbf{X}}^{\mathrm{new}}\in\Re^{n_{\mathrm{new}}\times I_{1}\times\ldots\times I_{N}}, \underline{\mathbf{Y}}^{\mathrm{new}}\in\Re^{n_{\mathrm{new}}\times J_{1}\times\ldots\times J_{M}}\right\}$   summary of previous data sets $\left\{\underline{\tilde{\mathbf{P}}}\in\Re^{F\times I_{1}\times\ldots\times I_{N}}, \underline{\tilde{\mathbf{Q}}}\in\Re^{F\times J_{1}\times\ldots\times J_{M}}\right\}$   centering parameters $\left\{\overline{\underline{\mathbf{X}}}\in\Re^{I_{1}\times\ldots\times I_{N}}, \overline{\underline{\mathbf{Y}}}\in\Re^{J_{1}\times\ldots\times J_{M}}\right\}$   number of points treated in the previous iteration$n^{\mathrm{old}}$  forgetting factor $\gamma$ number of factors $F$ *Outputs:* new summary of previous data sets $\left\{\underline{\tilde{\mathbf{P}}}^{\mathrm{new}}\in\Re^{F\times I_{1}\times\ldots\times I_{N}}, \underline{\tilde{\mathbf{Q}}}^{\mathrm{new}}\in\Re^{F\times J_{1}\times\ldots\times J_{M}}\right\}$   new centering parameters  $\left\{\overline{\underline{\mathbf{X}}}^{\mathrm{new}}\in\Re^{I_{1}\times\ldots\times I_{N}}, \overline{\underline{\mathbf{Y}}}^{\mathrm{new}}\in\Re^{J_{1}\times\ldots\times J_{M}}\right\}$, $n^{\mathrm{new}}$ prediction parameters  , , $\left\{\mathbf{b}_{f}\in\Re^{f}\right\}_{f=1}^{F}$  1. New centering parameters $\overline{\underline{\mathbf{X}}}^{\mathrm{new}}=\gamma\frac{n^{\mathrm{old}}}{n^{\mathrm{old}}+n^{\mathrm{new}}}\overline{\underline{\mathbf{X}}}+\left(1-\gamma\right)\frac{n^{\mathrm{new}}}{n^{\mathrm{old}}+n^{\mathrm{new}}}\text{Mean}_{1}\left(\underline{\mathbf{X}}^{\mathrm{new}}\right)$ $\overline{\underline{\mathbf{Y}}}^{\mathrm{new}}=\gamma\frac{n^{\mathrm{old}}}{n^{\mathrm{old}}+n^{\mathrm{new}}}\overline{\underline{\mathbf{Y}}}+\left(1-\gamma\right)\frac{n^{\mathrm{new}}}{n^{\mathrm{old}}+n^{\mathrm{new}}}\text{Mean}_{1}\left(\underline{\mathbf{Y}}^{\mathrm{new}}\right)$ $n^{\mathrm{new}}\leftarrow n^{\mathrm{old}}+n^{\mathrm{new}}$         1. Centering of the new data set $\left\{\underline{\mathbf{X}}^{\mathrm{new}},\underline{\mathbf{Y}}^{\mathrm{new}}\right\}$ along the first modality according to $\left\{\overline{\underline{\mathbf{X}}}^{\mathrm{new}},\overline{\underline{\mathbf{Y}}}^{\mathrm{new}}\right\}$ 2. Concatenating of the old and new data sets along the first modality   , $\underline{\mathbf{Y}}_{1}=\left[\begin{matrix} \gamma\underline{\tilde{\mathbf{Q}}} \\ \underline{\mathbf{Y}}^{\mathrm{new}} \end{matrix}\right]$  1. Pass over all factors   **for** $f=1$ to $F$  1. Initialization of the tensor $\underline{\mathbf{Y}}_{f}$ projectors   = $\left\{\mathbf{1}^{1},\ldots,\mathbf{1}^{M}\right\}$  1. **Iterate until the set** $\left\{\mathbf{q}_{f}^{1},\ldots,\mathbf{q}_{f}^{M}\right\}$ **converges** 2. Project $\underline{\mathbf{Y}}_{f}$ $\mathbf{u}_{f}=\underline{\mathbf{Y}}_{f}\times_{M+1}\mathbf{q}_{f}^{M}\times_{M}\ldots\times_{2}\mathbf{q}_{f}^{1}$     1. Determine $\left\{\mathbf{w}_{f}^{1},\ldots,\mathbf{w}_{f}^{N}\right\}$, $\left\Vert\mathbf{w}_{f}^{i}\right\Vert=1$ as decomposition of $\underline{\mathbf{Z}}_{f}=\underline{\mathbf{X}}_{f}\times_{1}\mathbf{u}_{f}$ 2. Project $\underline{\mathbf{X}}_{f}$ $\mathbf{t}_{f}=\underline{\mathbf{X}}_{f}\times_{M+1}\mathbf{w}_{f}^{N}\times_{N}\ldots\times_{2}\mathbf{w}_{f}^{1}$     1. Determine $\left\{\mathbf{q}_{f}^{1},\ldots,\mathbf{q}_{f}^{M}\right\}$ as decomposition of $\underline{\mathbf{R}}_{f}=\underline{\mathbf{Y}}_{f}\times_{1}\mathbf{t}_{f}$ 2. **end** $\mathbf{T}_{f}=\left[\mathbf{t}_{1}\vert\ldots\vert\mathbf{t}_{f}\right]$ $\mathbf{b}_{f}=\left(\mathbf{T}_{f}^{\mathrm{T}}\mathbf{T}_{f}\right)^{-1}\mathbf{T}_{f}^{\mathrm{T}}\mathbf{u}_{f}$ 5. **Data deflation:** $\underline{\mathbf{X}}_{f+1}=\underline{\mathbf{X}}_{f}-\mathbf{t}_{f}\circ\mathbf{w}_{f}^{1}\circ\ldots\circ\mathbf{w}_{f}^{N}$ $\underline{\mathbf{Y}}_{f+1}=\underline{\mathbf{Y}}_{f}-\left(\mathbf{T}_{f}\mathbf{b}_{f}\right)\circ\mathbf{q}_{f}^{1}\circ\ldots\circ\mathbf{q}_{f}^{M}$ 8. **end for** 9. **Matrix representation of data** 10. , 11. : , 13. **Matrix $\mathbf{T}_{F}$ orthogonalization** 14. : 15. , , 16. , 17. **Provide orthogonality of $\mathbf{T}_{F}^{\perp}$ and** $\mathbf{X}_{F+1}$ 18. **for** $f=1$ to $F$ $\tilde{\mathbf{p}}_{f}=\mathbf{p}_{f}+\mathbf{X}_{F+1}^{\mathrm{T}}\mathbf{t}_{f}^{\perp}$ 20. **end for** 21. , 22. **Tensor representation of the data** $\tilde{\mathbf{P}}^{\mathrm{new}}\in\Re^{F\times\left(I_{1}\cdot\ldots\cdot I_{N}\right)} \Rightarrow\underline{\tilde{\mathbf{P}}}^{\mathrm{new}}\in\Re^{F\times I_{1}\times\ldots\times I_{N}}$ $\tilde{\mathbf{Q}}^{\mathrm{new}}\in\Re^{F\times\left(J_{1}\cdot\ldots\cdot J_{M}\right)} \Rightarrow\underline{\tilde{\mathbf{Q}}}^{\mathrm{new}}\in\Re^{F\times J_{1}\times\ldots\times J_{M}}$ |
| a $\text{Mean}_{1}\left(\underline{\mathbf{X}}\right)$ is an average of the tensor $\underline{\mathbf{X}}$ along the first mode.  b The $n$-mode vector product of a tensor $\underline{\mathbf{X}}$ and vector $\mathbf{v}\in\Re^{I_{n}}$ is denoted $\underline{\mathbf{X}}\times_{n}\mathbf{v}$ [20].  c $\underline{\mathbf{X}}_{(i)}$ is the mode-$i$ unfolding of the tensor $\underline{\mathbf{X}}$ [20].  d $\text{vec}\left(\underline{\mathbf{X}}\right)$ is the vectorization of the tensor $\underline{\mathbf{X}}$ [20].  e The vector outer product is denoted as $\mathbf{w}\circ\mathbf{v}$ [20]. |
